# Occurrence and distribution of lost molars and furcation defects in a Bulgarian population: A retrospective three‐dimensional study

**DOI:** 10.1002/cre2.835

**Published:** 2024-02-03

**Authors:** Hristina Maynalovska, Antoaneta Mlachkova, Tsvetomil Voyslavov, Kamen Kotsilkov

**Affiliations:** ^1^ Department of Periodontology, Faculty of Dental Medicine Medical University of Sofia Sofia Bulgaria; ^2^ Department of Analytical Chemistry, Faculty of Chemistry and Pharmacy Sofia University St. Kliment Ohridski Sofia Bulgaria

**Keywords:** cone‐beam computed tomography, furcation defects, missing molars, tooth loss

## Abstract

**Objectives:**

To identify the ocuurrence, distribution, and factors associated with lost molars and furcation defects in a sample of the Bulgarian population.

**Material and Methods:**

The full mouth cone‐beam computed tomography of 56 male and 57 female patients, aged between 19 and 84 years, were examined. A comprehensive evaluation was performed on a total of 675 molars—339 in maxilla and 336 in mandible. Associations with variables such as age, gender, and periodontal disease were analyzed.

**Results:**

With aging the number of missing teeth and interradicular lesions increased. No significant links between gender and molar loss (*p* = .64) or gender and furcation involvement (*p* = .25) was found. Periodontitis was strongly associated with both studied dental problems (*p* < .001). The occurrence of furcation defects was more frequently observed in the maxilla than in the mandible.

**Conclusion:**

The occurrence and distribution of lost molars and furcation defects is substantial within the studied sample. Further investigation with a larger patient cohort is needed. Pertinent healthcare strategies to address the observed dental health issues also have to be developed.

## 1 INTRODUCTION

Tooth loss is a valuable indicator of the oral health within a population. It is a complex outcome of an individual's dental history—attitude, diseases, and treatments over the course of life. The number of missing teeth exerts a profound influence on overall quality of life, as it can lead to masticatory dysfunction. This, in turn, contributes to compromised nutrition, poor general health, diminished quality of life and low self‐esteem. The socioeconomic impact of tooth loss is substantial and significantly escalates healthcare costs (Kassebaum et al., 2014).

A multitude of variables have been linked to tooth loss, but a consensus remains elusive regarding whether dental disease‐related factors or socio‐behavioral factors bear the greatest significance as risk indicators (Müller et al., 2007). Some investigators have identified periodontitis as the most prevalent cause of tooth extraction among individuals aged over 40 years (Phipps & Stevens, 1995; Reich & Hiller, 1993). The World Health Organization has identified periodontitis as a major contributor to tooth loss in the global adult population. Furthermore, the burden of periodontal diseases continues to be substantial. Severe periodontal disease exhibits a global prevalence of approximately 19% in individuals aged 15 years or older, encompassing over 1 billion cases worldwide (Global oral health status report, 2022).

The progression of periodontitis leads to the involvement of the furcation area of the posterior teeth by the periodontal infection. The intricate anatomy of molars, outlined with the complex morphology of the furcation area, contributes to the development of more severe disease and a less favorable long‐term prognosis for these teeth (Wang et al., 1994). Some researches have even hypothesized genetic factors which may influence the risk of furcation lesion development (Pashova–Tasseva, 2021). A systematic review suggests that furcation involvement doubles the risk of tooth loss in molars (Nibali et al., 2016).

Detecting and accurately estimating the degree of furcation involvement are of keystone importance when determining a treatment plan and deciding whether to preserve or extract a tooth. The routine diagnosis of furcation involvement relies on clinical probing with a curved periodontal probe and two‐dimensional radiographic imaging. However, in many cases, these methods do not offer sufficient information regarding the periodontal support of the affected tooth, especially concerning the interradicular bone (Müller & Eger, 1999; Zhang et al., 2018).

In contemporary practice, the utilization of cone‐beam computed tomography (CBCT) represents a significant advancement in the precision of furcation diagnosis. This method greatly enhances the ability to accurately assess and classify furcation involvement. Additionally, CBCT allows for the identification of morphological variations and pathological findings, such as root trunk length, the degree of root separation, fusion of adjacent roots, reduced interradicular space, concurrent periapical pathology, the presence of combined periodontal‐endodontic lesions, root perforation, fenestration, and insufficient root canal obturation (Qiao et al., 2014; Scarfe et al., 2017; Walter et al., 2010).

The primary objective of every dental therapy is to prevent tooth loss, which, from the patient's perspective, is the most significant treatment outcome. To deliver appropriate dental care to individual patients, a comprehensive diagnostic and prognostic risk assessment is essential, alongside access to effective and evidence‐based treatment modalities. To optimize oral healthcare strategies, it is imperative to conduct a thorough assessment of the existing dental issues (Petersen & Yamamoto, 2005).

Existing information on the prevalence and distribution of lost molars and furcation involvement among individuals is limited. A comprehensive understanding of current trends is indispensable for the planning of dental services. In a study involving a sample of 222 periodontal patients, Svadström et al. reported that 3% of the participants had lost all of their molar teeth (Svärdström & Wennström, 1996). Among patients aged over 40 years, every second molar exhibited advanced periodontal destruction in the furcation area. Albender et al. reported a prevalence of furcation defects at 13.7% in the general population in the United States (Albandar et al., 1999). In a study conducted by Najim et al. (2016), which involved 329 subjects, the prevalence of molars with furcation involvement was found to be 8.3%.

The **aim** of the current research was to analyze the status in a sample of the Bulgarian population—to identify the ocuurrence, distribution of lost molars and furcation defect and to outline association with variables like age, gender, and periodontitis. The study was based on cone‐beam computed tomography images as they have been accepted as relevant and detailed paraclinical source of information.

## 2 METHODS

This is a three‐dimensional retrospective radiographic study that examines the occurrence and distribution of lost molars and furcation defects in a convenience sample of individuals who had undergone full‐mouth scans during their dental treatment for various dental issues. These scans were performed in accordance with European CBCT guidelines as part of the patients’ ongoing dental treatment. Exclusion criteria consisted of individuals under 18 years and CBCT scans with compromised diagnostic quality.

The scans were randomly chosen from a list of all patients with available CBCTs in the database of a private dental clinic. Each patient was assigned a unique numerical identifier. The initial sample size was determined at 100. A random number generator (Microsoft Excell) created a set of 100 random numbers, covering the range of numerical identifiers assigned to patients in the sampling frame. The selected CBCT scans were divided into five age groups: up to 29 years, 30–39 years old, 40–49 years old, 50–59, and over 60 years old. To ensure balanced representation across age subgroups, 13 more scans were added by similar randomization. Therefore, the number of the analyzed CBCT was 113.

A total sample of 675 molar teeth were explored—339 were in the maxilla and 336—in the mandible. All molars except wisdom teeth were included. Root remnants have been considered as missing. Dental implants have also been excluded from the research.

Ethical approval for the study was obtained from the Research Ethics Committee at the Medical University of Sofia—KENIMUS (Protocol No. 02/24.02.2023). The research was carried out in compliance with the Helsinki Declaration. All participants were informed about the design and the aim of the study and signed an informed consent.

The CBCTs were performed with Planmeca Promax 3D Mid, settings at 90 kV, 6 mA, and voxel size 200 µm, field of view 10 × 10 cm^2^. The 3D images of each molar tooth were analyzed in the horizontal, sagittal, and transversal section by one investigator. The software Planmeca Romexis Viewer 5.3.5.80 with digital measurement tools was used.

**Figure 1:**
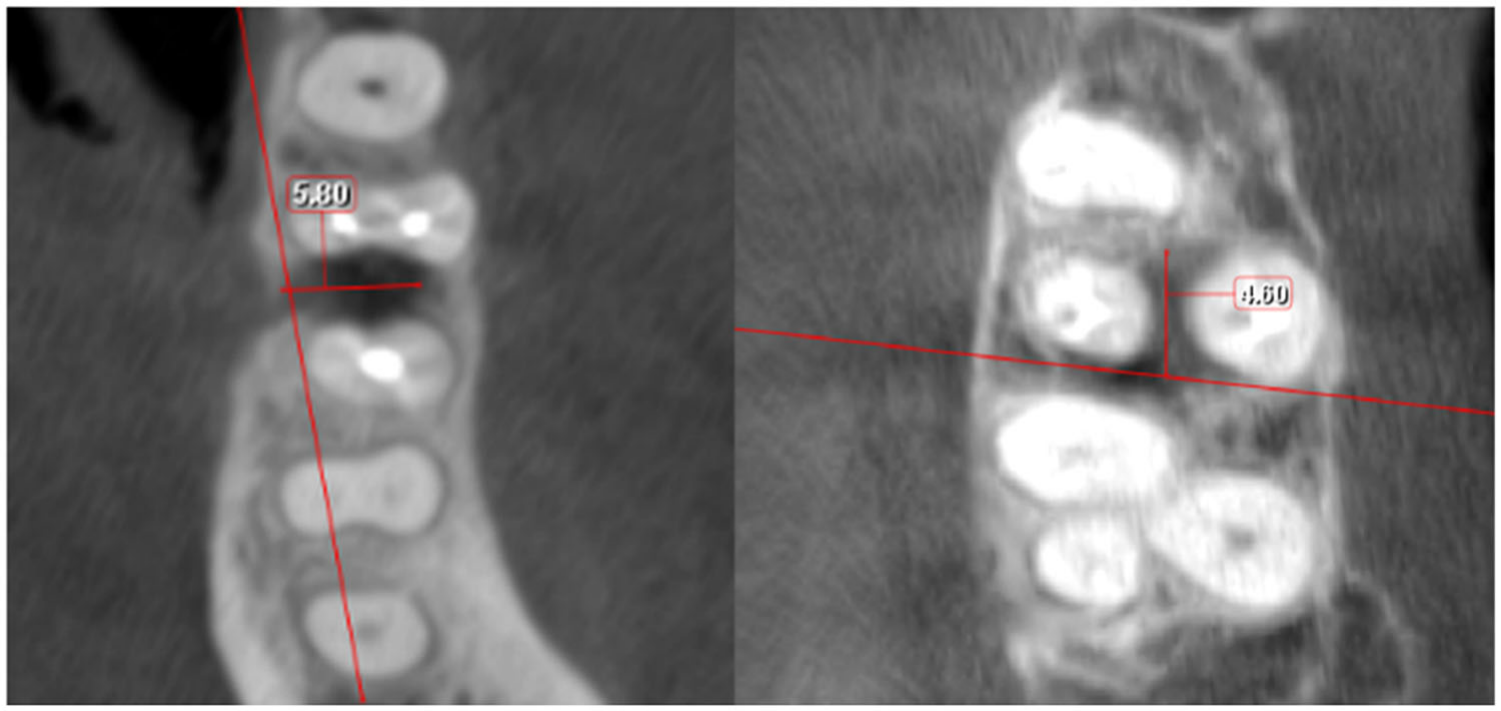
Radiographic assessment of the degree of furcation involvement.

The depth of the furcation defects was registered in the horizontal plane by measuring the distance in millimeters between the outer root surfaces of the tooth roots adjacent to the defect and the inter‐radicular bone (Figure 1).

To assess the extent of the furcation lesion Hamp et al.'s (1975) classification was employed. Although designed for clinical evaluation, focusing on the horizontal loss of periodontal tissue support, Hamp's classification was correlated with the radiographic bone loss in the furcation area as follows:

Degree I: horizontal radiographic bone loss up to 3 mm.

Degree II: horizontal radiographic bone loss exceeding 3 mm, but no “through and through” destruction.

Degree III: horizontal “through and through” destruction in the furcation.

Furcations were considered healthy if the furcation area was filled with bone up to the fornix and no radiolucency between the tooth roots was observed.

The radiographic diagnosis of periodontitis was based on the assessment of marginal alveolar bone loss, a key secondary indicator of periodontitis (Tonetti et al., 2018). Patients were categorized as “healthy” when the interproximal bone level was at the physiological height (with a distance between the cemento‐enamel junction and the alveolar crest measuring up to 3 mm). Those with “periodontitis” were identified when generalized interproximal bone loss was evident (with a distance exceeding 3 mm between the cemento‐enamel junction and the alveolar crest) (Goodarzi Pour et al., 2015; Goodson et al., 1984).

The raw data were compiled into a master Excel spreadsheet, and subsequent data coding was carried out for statistical analysis. Association between the studied variables (lost molars and furcation defects) and factors such as age, gender and periodontal disease were assessed using the Chi‐square test and analysis of variance (ANOVA). The null hypothesis was rejected at *p* < .05. To identify outliers that deviated significantly from the overall data pattern, the Interquartile Range method was employed. A Student's *t*‐test was used to compare the mean number of lost molars per person between two groups.

All statistical analyses were performed using Microsoft Excel.

## 3 RESULTS

The study included a population of 113 individuals, comprising 56 males and 57 females, with ages ranging from 19 to 84 years (*M* = 45.50 years, *SD* = 14.25). Approximately 41% of the patients in the study had all their molar teeth present, while nearly 3% had lost all of them. The sample of teeth included 675 first and second molars, with 339 in the maxilla and 336 in the mandible. In the analyzed patient sample, 25% of the molars of interest (first and second molars) had been lost.

Table 1 provides an overview of the study population's distribution by age and gender, along with the the number of remaining molars in various age groups and across gender categories.

**Table 1:** Distribution of the study population and remaining molars according to age and gender.

Age		Up to 29	30–39	40–49	50–59	Over 60
**Number of patients**		**10**	**10**	**21**	**8**	**7**
Male						
Number of present molars	8	9	7	5	3	0
	7	1	1	2	0	0
	6	0	1	4	2	0
	5	0	0	3	0	3
	4	0	1	4	1	1
	3	0	0	1	0	2
	2	0	0	0	1	1
	1	0	0	1	0	0
	0	0	0	1	1	0
**Number of patients**		**10**	**10**	**9**	**15**	**13**
Female						
Number of present molars	8	7	5	4	4	2
	7	1	1	2	2	2
	6	2	2	1	0	2
	5	0	2	1	4	1
	4	0	0	1	0	1
	3	0	0	0	2	1
	2	0	0	0	2	2
	1	0	0	0	1	1
	0	0	0	0	0	1

In the group of patients aged up to 29 years, 80% had all eight molars, while in the 30–39 age group, only 60% retained all their distal teeth. For the 40–49 and 50–59 age groups, only one‐third of individuals had all eight molars. In contrast, just 6% of those aged 60 or older had all their molar teeth preserved, with an equal number of patients in this age group having lost all first and second molars.

**Figure 2:**
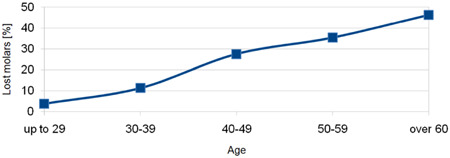
Age related to molar loss.

A Chi‐square test of independence was performed to analyze the association between the number of lost molars and gender. The results indicated no significant association between gender and molar loss (*χ* ^2^_(1, *N* = 113)_ = 0.2125, *p* = .64). Similarly, was assessed the relationship between age and the lost molars. This analysis revealed a significant relationship (*χ* ^2^_(4, *N* = 113)_ = 26.1212, *p* = .00003). Older individuals were more likely to have lost a molar compared to younger individuals (Figure 2).

**Figure 3:**
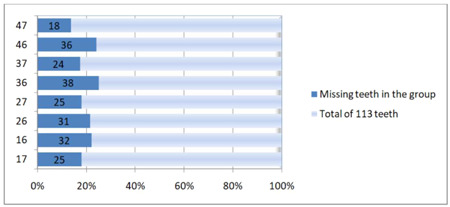
Distribution of lost molars by tooth position.

Figure 3 displays the distribution of lost molars based on tooth position. To determine whether there was a tendency for a particular molar to be most commonly or least commonly missing, an analysis was conducted using the interquartile range method. The findings revealed that no values fell beyond the upper and lower fences of the Interquartile Range. As a result, there were no statistically significant differences in molar loss based on tooth position.

**Figure 4:**
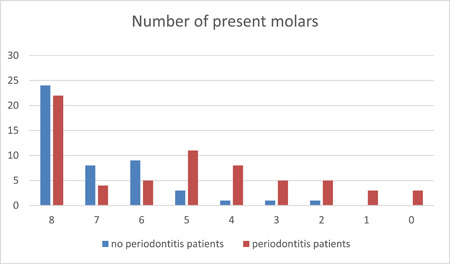
Distribution of remaining molars in periodontitis and non‐periodontitis patients.

Out of the individuals included in the study, 66 were diagnosed with periodontitis based on radiographic findings, while 47 did not exhibit radiographic signs of periodontal disease. Figure 4 illustrates the number of remaining molars in these two patient groups.

The average number of lost molars per person in the periodontitis patient group was 2.71, which is 2.56 times greater than the mean number (1.06) in the non‐periodontitis group. It was hypothesized that the molar loss in the periodontitis group (*M* = 50.85%, *SD* = 24.62, *n* = 44) exceeded the molar loss in the No Periodontitis group (*M* = 27.17%, *SD* = 16.71, *n* = 23). This difference was statistically significant, with *p* < .001.

Out of the 675 molar teeth in the sample, 205 (30%) exhibited at least one furcation defect—119 in the maxilla and 86 in the mandible. Table 2 provides details on the distribution of furcation defects by tooth position, age, and gender.

**Table 2:** Distribution of furcation defects by tooth position, age, and gender.

	Tooth	Up to 29	30–39	40–49	50–59	Over 60
Male	17	0	4	9	4	1
	16	0	4	5	2	3
	26	0	4	6	2	3
	27	0	3	3	2	1
	36	0	1	6	4	3
	37	0	2	5	1	1
	46	0	1	4	9	1
	47	1	0	4	10	2
Female	17	0	5	6	4	3
	16	1	1	4	5	6
	26	0	0	5	5	3
	27	0	2	6	4	3
	36	0	2	1	3	3
	37	0	1	4	6	2
	46	0	0	2	0	1
	47	1	1	3	0	1

To examine the relationship between age and furcation involvement and to assess the influence of gender, a two‐way ANOVA was conducted. The analysis did not reveal a statistically significant difference in means between groups based on gender (*F*_(1)_ = 1.267, *p* = .25), with the *p* value exceeding .05. As a result, the null hypothesis could not be rejected.

However, the results concerning the second factor, age (*F*_(2)_ = 15.146, *p* = .001), indicated a *p* value lower than .05. In this case, the null hypothesis was rejected, signifying a statistically significant influence of age on furcation prevalence.

Table 3 presents the distribution of furcation defects and their severity concerning tooth position. To assess disparities in furcation involvement across the maxilla, mandible, and both jaws combined, we applied the Interquartile Range method. Upon analysis, no statistically significant differences in furcation involvement were observed based on tooth position in the maxilla, mandible, or when considering all eight molars collectively.

**Table 3:** Number of molars concerning jaw, position and severety of furcation involvement (Degree I–III).

	Maxilla				Mandible				Total
Furcation involvement	17	16	26	27	36	37	46	47	
Without FI	52	50	54	64	52	67	59	72	**470**
I	14	7	6	6	3	1	3	6	**46**
II	7	11	9	12	8	8	7	9	**71**
III	15	13	13	6	11	14	8	8	**88**
Total with FI	**36**	**31**	**28**	**24**	**22**	**23**	**18**	**23**	**205**
Total	**88**	**81**	**82**	**88**	**74**	**90**	**77**	**95**	**675**

**Figure 5:**
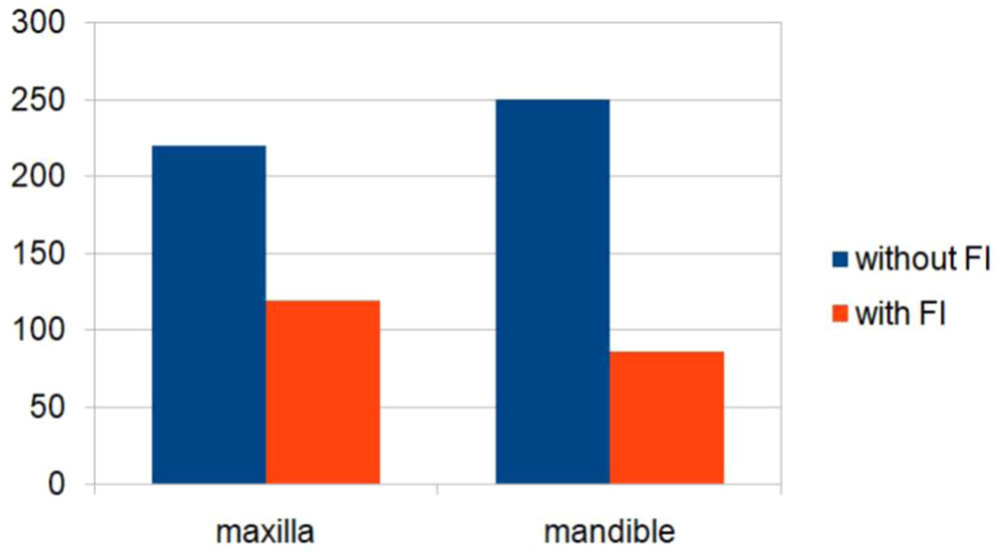
Distribution of furcation involved teeth in maxilla and mandible.

A Chi‐square test of independence was conducted to investigate the relationship between the jaw and the presence of furcation defects. The analysis showed a significant relationship between these variables, *χ* ^2^_(1, *N* = 675)_ = 7.21, *p* = .007. Notably, maxillary molars were more prone to exhibit furcation defects compared to mandibular molars (see Figure 5).

Out of the affected molars, 181 (88%) were observed in patients with periodontitis. Within this group, only 18% of the patients had molars without furcation defects. In contrast, among the patients without radiographic signs of periodontal disease, 65% had molars without furcation defects. The association between furcation involvement and periodontal disease exhibited strong statistical significance (*χ* ^2^_(1, *N* = 113)_ = 26.58, *p* < .001).

## 4 DISCUSSION

Poor oral health has a direct impact on overall health and can significantly affect a person's quality of life. A valuable marker for assessing the level of oral health within a specific population is the occurrence of tooth loss. It reflects the occurrence of dental diseases, the perspectives of individuals toward oral care, as well as the attitudes of both dentists and patients regarding available treatment options and the accessibility of dental services (Baelum et al., 2007; Petersen et al., 2005). According to a study the decision to extract a tooth instead of preserving it is usually a consequence of two key factors: the absence of alternative treatment options at the time of the dental appointment and the prohibitively high cost associated with the procedure required to safe the tooth (Silva‐Junior et al., 2017).

First and second molars are the most frequently lost teeth in the dentition van der Velden et al., 2015). Withinn the studied sample, it was observed that 70% of the patients over the age of 40 had lost at least one molar tooth, and only 6% of the individuals over 60 retained all eight molars. There were no discernible gender differences in tooth loss. This finding aligns with the research by Kassebaum et al. (2014), which indicated a global reduction in gender‐based differences in tooth loss between 1990 and 2010. It's plausible that nowadays, both men and women share similar attitudes towards oral hygiene, dental treatment, and have equal access to dental care.

In the studied population, a strong association between tooth loss and age was evident, a finding consistent with the cumulative impact of oral diseases over the course of one's life and in alignment with prior reports (Fatima Del Carmen et al., 2021; Gabiec et al., 2022; Kassebaum et al., 2014). Statistically significant differences in the molar loss based on tooth position were also searched but such distinctions were not substantiated.

The causes of tooth loss are various and widely discussed in the literature. Periodontitis is often recognized as a primary contributor to tooth loss (Global oral health status report, 2022; Neely et al., 2005; Phipps & Stevens, 1995; Reich & Hiller, 1993). This current study reaffirmed the strong association between the number of lost molars and the presence of periodontal disease.

Frequently, the infection‐related involvement of the furcation area in molar teeth is considered a risk factor for tooth loss (Nibali et al., 2016). In the studied sample, the number of teeth with furcation involvement increased with age, a pattern consistent with the majority of epidemiological surveys that have demonstrated a positive association between aging and the severity of periodontal disease. No statistically significant association between gender and interradicular defects was proved, a result in line with Najim et al. 2016 (Najim et al., 2016) who also confirmed that gender has no association with the presence of furcation defects.

Most epidemiologic studies identify the first upper molars as the most frequently affected by bone destruction in the furcation area (Neely et al., 2005; Sachdeva et al., 2020). However, in our sample, no statistically significant differences in the furcation involvement by tooth position were observed. This discrepancy may be attributed to the fact that the majority of research on furcation defects’ prevalence analyzes samples of individuals diagnosed with periodontitis. In our study, a notable proportion—almost 42% of individuals—lacked radiographic signs of periodontal disease, and 35% were under the age of 40.

The results indicated that bone loss in the furcation area was more frequently observed in the maxilla than in the mandible, consistent with the findings of Najim et al. (2016) and Svardstom and Wennström (1996). This contrasts with the observations made by Sachdeva et al. (2020). One possible explanation for the statistically significant higher involvement of the upper molars can be attributed to the morphological characteristics of maxillary teeth. The trifurcation area, where two of the furcation entrances are situated on the interproximal surfaces, is often associated with elevated plaque levels and challenges in plaque control. These factors contribute to the increased severity of periodontal destruction. It's worth noting that diagnosing furcation involvement is often a complex process, and lesions in this area are frequently underestimated and/or missed by clinicians.

This study presents several limitations. Notably, the relatively small sample size is a key consideration, owing to the fact that 3D imaging is not a routine paraclinical diagnostic tool in dentistry. Furthermore, this analysis was primarily driven by radiographic findings, with limited exploration of potential associations with other clinical and paraclinical variables. Future research should aim to investigate the relationships between tooth position, tooth morphological characteristics, and the severity of furcation involvement. Examining these relationships and interactions could greatly enhance treatment planning by incorporating tooth‐related factors into today's evidence‐based knowledge.

The strength of this study lies in the sample selection, which encompassed both patients with and without periodontitis. To the best of our knowledge, this study represents the first epidemiological investigation of furcation defect occurrence and distribution based on three‐dimensional imaging.

## 5 CONCLUSION

In conclusion, the occurrence and distribution of lost molars and furcation defects were found to be notably high within our studied sample. The research revealed that as individuals age, the number of missing teeth and teeth with interradicular lesions tends to increase, with no discernible gender differences in this trend. Additionally, a robust association was established between periodontitis and the occurrence of both lost molars and furcation defects.

These findings have important implications for dental care and public health. They can serve as a foundation for more effective preventive strategies customized to individual needs, promoting risk profiling, early diagnosis, and timely treatment.

Further investigations with representative samples are needed.

## AUTHOR CONTRIBUTIONS

**Hristina Maynalovska**: substantially contributed to the conception, design, data acquisition, analysis, interpretation of the research and in writing and editing the manuscript. **Antoaneta Mlachkova**: contributed to the conception of the study and the interpretation of data, and critically revised the manuscript. **Tsvetomil Voyslavov**: was involved in the design of the research, participated in the analysis and interpretation of data and in writing the manuscript. **Kamen Kotsilkov**: made significant contributions to the conception of the study, participated in data acquisition and analysis, and critically revised the manuscript. All authors gave their final approval of the version to be published.

## CONFLICT OF INTEREST STATEMENT

The authors declare no conflicts of interest.

## Data Availability

The data that support the findings of this study are available from the corresponding author upon reasonable request. The data that support the findings of this study are available on request from the corresponding author (Maynalovska, Hr.).

## References

[cre2835-bib-0001] Albandar, J. M. , Brunelle, J. A. , & Kingman, A. (1999). Destructive periodontal disease in adults 30 years of age and older in the United States, 1988‐1994. Journal of Periodontology, 70(1), 13–29. 10.1902/jop.1999.70.1.13 10052767

[cre2835-bib-0002] Baelum, V. , van Palenstein Helderman, W. , Hugoson, A. , Yee, R. , & Fejerskov, O. (2007). A global perspective on changes in the burden of caries and periodontitis: Implications for dentistry. Journal of Oral Rehabilitation, 34, 872–906. 18034671 10.1111/j.1365-2842.2007.01799.x

[cre2835-bib-0004] Fatima Del Carmen, A. D. , Aída, B. Y. S. , & Javier, F. H. (2021). Risk indicators of tooth loss among Mexican adult population: A cross‐sectional study. International Dental Journal, 71, 414–419. 33642043 10.1016/j.identj.2020.12.016 PMC9275087

[cre2835-bib-0006] Gabiec, K. , Bagińska, J. , Łaguna, W. , Rodakowska, E. , Kamińska, I. , Stachurska, Z. , Dubatówka, M. , Kondraciuk, M. , & Kamiński, K. A. (2022). Factors associated with tooth loss in general population of Bialystok, Poland. International Journal of Environmental Research and Public Health, 19, 2369. 10.3390/ijerph19042369 35206557 PMC8872086

[cre2835-bib-0007] Global oral health status report : Towards universal health coverage for oral health by 2030. Geneva: World Health Organization; 2022. Licence: CC BY‐NC‐SA 3.0 IGO.

[cre2835-bib-0008] Goodarzi Pour, D. , Romoozi, E. , & Soleimani Shayesteh, Y. (2015). Accuracy of cone beam computed tomography for detection of bone loss. Journal of Dentistry (Tehran, Iran), Jul 12(7), 513–523. 26877741 PMC4749417

[cre2835-bib-0009] Goodson, J. M. , Haffajee, A. D. , & Socransky, S. S. (1984). The relationship between attachment level loss and alveolar bone loss. Journal of Clinical Periodontology, 11, 348–359. 10.1111/j.1600-051X.1984.tb01331.x 6585374

[cre2835-bib-0010] Hamp, S.‐E. , Nyman, S. , & Lindhe, J. (1975). Periodontal treatment of multi rooted teeth. Journal of Clinical Periodontology, 2, 126–135. 10.1111/j.1600-051X.1975.tb01734.x 1058213

[cre2835-bib-0011] Kassebaum, N. J. , Bernabé, E. , Dahiya, M. , Bhandari, B. , Murray, C. J. L. , & Marcenes, W. (2014). Global burden of severe tooth loss: A systematic review and meta‐analysis. Journal of Dental Research, 93(7 Suppl.), 20S–28S. 10.1177/0022034514537828. 24947899 PMC4293725

[cre2835-bib-0012] Müller, F. , Naharro, M. , & Carlsson, G. E. (2007). What are the prevalence and incidence of tooth loss in the adult and elderly population in Europe? Clinical Oral Implants Research, 18(Suppl. 3), 2–14. 10.1111/j.1600-0501.2007.01459.x 17594365

[cre2835-bib-0013] Müller, H. P. , & Eger, T. (1999). Furcation diagnosis. Journal of Clinical Periodontology, 26(8), 485–498. 10.1034/j.1600-051x.1999.260801.x 10450808

[cre2835-bib-0014] Najim, U. , Slotte, C. , & Norderyd, O. (2016). Prevalence of furcation‐involved molars in a Swedish adult population. A radiographic epidemiological study. Clinical and Experimental Dental Research, 2, 104–111. 10.1002/cre2.27 29744156 PMC5839259

[cre2835-bib-0015] Neely, A. L. , Holford, T. R. , Löe, H. , Ånerud, Å. , & Boysen, H. (2005). The natural history of periodontal disease in humans: Risk factors for tooth loss in caries‐free subjects receiving no oral health care. Journal of Clinical Periodontology, 32, 984–993. 16104963 10.1111/j.1600-051X.2005.00797.x

[cre2835-bib-0016] Nibali, L. , Zavattini, A. , Nagata, K. , Di Iorio, A. , Lin, G.‐H. , Needleman, I. , & Donos, N. (2016). Tooth loss in molars with and without furcation involvement—A systematic review and meta‐analysis. Journal of Clinical Periodontology, 43, 156–166. 10.1111/jcpe.12497 26932323

[cre2835-bib-0018] Pashova –Tasseva, Z. (2021). Significance of gene polymorphism in severe periodontitis. Dissertation, Medical University‐Sofia, 88.

[cre2835-bib-0020] Petersen, P. E. , Bourgeois, D. , Ogawa, H. , Estupinan‐Day, S. , & Ndiaye, C. (2005). The global burden of oral diseases and risks to oral health. Bulletin of the World Health Organization, 83, 661–669. [PMC free article] [PubMed] [Google Scholar]. 16211157 PMC2626328

[cre2835-bib-0021] Petersen, P. E. , & Yamamoto, T. (2005). Improving the oral health of older people: The approach of the WHO global oral health programme. Community Dentistry and Oral Epidemiology, 33, 81–92. 10.1111/j.1600-0528.2004.00219.x 15725170

[cre2835-bib-0022] Phipps, K. R. , & Stevens, V. J. (1995). Relative contribution of caries and periodontal disease in adult tooth loss for an HMO dental population. Journal of Public Health Dentistry, 55(4), 250–252. 10.1111/j.1752-7325.1995.tb02377.x 8551465

[cre2835-bib-0023] Qiao, J. , Wang, S. , Duan, J. , Zhang, Y. , Qiu, Y. , Sun, C. , & Liu, D. (2014). The accuracy of cone‐beam computed tomography in assessing maxillary molar furcation involvement. Journal of Clinical Periodontology, 41, 269–274. 10.1111/jcpe.12150 24372315

[cre2835-bib-0024] Reich, E. , & Hiller, K.‐A. (1993). Reasons for tooth extraction in the western states of Germany. Community Dentistry and Oral Epidemiology, 21, 379–383. 10.1111/j.1600-0528.1993.tb01103.x 8306617

[cre2835-bib-0025] Sachdeva, S. , Mani, A. , Saluja, H. , Phadnaik, M. B. , & Singh, M. (2020). Prevalence and distribution of bone defects associated with moderate and severe periodontitis patients. Clinical Epidemiology and Global Health, 8(3), 712–717.

[cre2835-bib-0026] Scarfe, W. C. , Azevedo, B. , Pinheiro, L. R. , Priaminiarti, M. , & Sales, M. A. O. (2017). The emerging role of maxillofacial radiology in the diagnosis and management of patients with complex periodontitis. Periodontology 2000, 74, 116–139. 10.1111/prd.12193 28429477

[cre2835-bib-0027] Silva‐Junior, M. F. , Sousa, A. C. C. , Batista, M. J. , & Sousa, M. L. R. (2017). Condição de saúde bucal e motivos para extração dentária entre uma população de adultos (20‐64 anos). Ciência & Saúde Coletiva, 22(8), 2693–2702. 10.1590/1413-81232017228.22212015 28793083

[cre2835-bib-0028] Svärdström, G. , & Wennström, J. L. (1996). Prevalence of furcation involvements in patients referred for periodontal treatment. Journal of Clinical Periodontology, 23, 1093–1099. 8997653 10.1111/j.1600-051x.1996.tb01809.x

[cre2835-bib-0029] Tonetti, M. S. , Greenwell, H. , & Kornman, K. S. (2018). Staging and grading of periodontitis: Framework and proposal of a new classification and case definition. Journal of Periodontology, 89(Suppl. 1), S159–S172. 10.1002/JPER.18-0006 29926952

[cre2835-bib-0031] van der Velden, U. , Amaliya, A. , Loos, B. G. , Timmerman, M. F. , van der Weijden, F. A. , Winkel, E. G. , & Abbas, F. (2015). Java project on periodontal diseases: Causes of tooth loss in a cohort of untreated individuals. Journal of Clinical Periodontology, 42, 824–831. 10.1111/jcpe.12446 26269207

[cre2835-bib-0032] Walter, C. , Weiger, R. , & Zitzmann, N. U. (2010). Accuracy of three‐dimensional imaging in assessing maxillary molar furcation involvement. Journal of Clinical Periodontology, 37, 436–441. 10.1111/j.1600-051X.2010.01556.x 20374414

[cre2835-bib-0033] Wang, H. L. , Burgett, F. G. , Shyr, Y. , & Ramfjord, S. (1994). The influence of molar furcation involvement and mobility on future clinical periodontal attachment loss. Journal of Periodontology, 65(1), 25–29. 10.1902/jop.1994.65.1.25 8133412

[cre2835-bib-0034] Zhang, W. , Foss, K. , & Wang, B. Y. (2018). A retrospective study on molar furcation assessment via clinical detection, intraoral radiography and cone beam computed tomography. BMC Oral Health, 18(1), 75. 10.1186/s12903-018-0544-0 29724208 PMC5934848

